# Living alone with dementia in Japan: challenges and policies

**DOI:** 10.1002/alz.087607

**Published:** 2025-01-09

**Authors:** Shuichi Awata

**Affiliations:** ^1^ Tokyo Metropolitan Institute for Geriatrics and Gerontology, Tokyo Japan

## Abstract

**Background:**

Due to declining birthrate and increasing longevity, the number of older people living alone with cognitive impairment is rapidly increasing in Japan. They have a lot of challenges in terms of health, housing, finance, daily life and protection of rights, all of which should be clarified to create inclusive, equitable, and sustainable super‐aged societies.

**Methods:**

A series of factual investigation was conducted using existing statical materials, community‐based epidemiological studies, clinic‐based case studies, and literature reviews.

**Results:**

According to the national prevalence rate of dementia and the future population estimate by household type, the percentage of older people living alone with dementia were estimated to increase with age, particularly in female; those in oldest‐old population aged 85 years and more were estimated to be 7% in male and 14% in female in 2025 and rapidly increase during the first half of 21th century. A series of factual investigations suggested that older people living alone with cognitive impairment were more likely to feel lonely, have poor nutritional status, become victims of fraud and natural disasters, die after missing, and experience disruption of continuous living in a community. “Community Space”, the places where older people are able to stay freely and comfortably in a community, may contribute to improve accessibility to health and social care services. In addition to public advocacy systems such as the adult gurdian system, every service provider, prticularly health and social care service providers, should learn the skill for decision support. Public‐private partnership should be promoted to prevail rational accommodation in social environment.

**Conclusion:**

The national and local governments should formulate comprehensive policies to ensure human rights of older people living alone with cognitive impairment in a community. The first version of the guidance to create social environment to support the lives of older people living alone with cognitive impairment was published in Japan.

